# Breast cancer colonization by *Fusobacterium nucleatum* accelerates tumor growth and metastatic progression

**DOI:** 10.1038/s41467-020-16967-2

**Published:** 2020-06-26

**Authors:** Lishay Parhi, Tamar Alon-Maimon, Asaf Sol, Deborah Nejman, Amjad Shhadeh, Tanya Fainsod-Levi, Olga Yajuk, Batya Isaacson, Jawad Abed, Naseem Maalouf, Aviram Nissan, Judith Sandbank, Einav Yehuda-Shnaidman, Falk Ponath, Jörg Vogel, Ofer Mandelboim, Zvi Granot, Ravid Straussman, Gilad Bachrach

**Affiliations:** 1 The Institute of Dental Sciences, The Hebrew University-Hadassah School of Dental Medicine, Jerusalem, Israel; 2 Department of Molecular Cell Biology, Weizmann Institute of Science, Rehovot, Israel; 3 Department of Developmental Biology and Cancer Research, Institute for Medical Research Israel Canada (IMRIC), Hebrew University-Hadassah Medical School, Jerusalem, Israel; 4 Department of Immunology and Cancer Research, Institute for Medical Research Israel Canada (IMRIC), Hebrew University-Hadassah Medical School, Jerusalem, Israel; 5 Department of General and Oncological Surgery-Surgery C, The Chaim Sheba Medical Center, Tel Hashomer, Ramat Gan, Israel; 6 The Pathology Institute, Maccabi Healthcare Services, Rehovot, Israel; 7 Helmholtz Institute for RNA-based Infection Research (HIRI), Helmholtz Center for Infection Research (HZI), Würzburg, Germany; 8 Institute for Molecular Infection Biology, Medical Faculty, University of Würzburg, Würzburg, Germany

**Keywords:** Breast cancer, Bacteriology

## Abstract

*Fusobacterium nucleatum* is an oral anaerobe recently found to be prevalent in human colorectal cancer (CRC) where it is associated with poor treatment outcome. In mice, hematogenous *F. nucleatum* can colonize CRC tissue using its lectin Fap2, which attaches to tumor-displayed Gal-GalNAc. Here, we show that Gal-GalNAc levels increase as human breast cancer progresses, and that occurrence of *F. nucleatum* gDNA in breast cancer samples correlates with high Gal-GalNAc levels. We demonstrate Fap2-dependent binding of the bacterium to breast cancer samples, which is inhibited by GalNAc. Intravascularly inoculated Fap2-expressing *F. nucleatum* ATCC 23726 specifically colonize mice mammary tumors, whereas Fap2-deficient bacteria are impaired in tumor colonization. Inoculation with *F. nucleatum* suppresses accumulation of tumor infiltrating T cells and promotes tumor growth and metastatic progression, the latter two of which can be counteracted by antibiotic treatment. Thus, targeting *F. nucleatum* or Fap2 might be beneficial during treatment of breast cancer.

## Abstract (web summary)

High levels of *Fusobacterium nucleatum* have been associated with poor overall survival in patients with colorectal and esophageal cancer. Here, the authors show that *F. nucleatum* is abundant in breast cancer samples and that the colonization by *F. nucleatum* accelerates tumor growth and metastasis in preclinical breast cancer models.

## Introduction

*Fusobacterium nucleatum* is a common oral non-spore forming gram-negative anaerobe that has long been known to be associated with the development of periodontal disease. More recently, however, genomic studies provided evidence that *F. nucleatum* may also be prevalent in colorectal carcinoma^1,2^. In support of this proposed link with cancer, subsequent studies demonstrated that *F. nucleatum* promotes tumorigenesis^3,4^, affects infiltration of tumor-infiltrating lymphocytes^5–7^, inhibits killing of cancer cells by Natural Killer (NK) cells and tumor-infiltrating T cells^8^, and induces resistance to chemotherapy in colon cancer^9,10^. Moreover, treatment of mice bearing a colon cancer xenograft with antibiotics to eliminate *F. nucleatum* concomitantly reduced cancer cell proliferation and overall tumor growth^11^. Together, these CRC-related fusobacterial effects provide a rational for the observation that high numbers of *F. nucleatum* in CRC tissue is inversely correlated with overall survival^12^ (also reviewed in ref. ^13^). In addition to CRC, a high burden of *F. nucleatum* is also associated with poor prognosis in esophageal cancer^14,15^.

CRC-associated fusobacteria originate from the oral microbial community, and we have previously hypothesized that they reach the colon via the hematogenous rather than the gastrointestinal route^16^. In this model, *F. nucleatum* will enter the bloodstream during transient bacteremia, which is frequent in periodontal disease, and specifically dock to CRC tissue through its surface-exposed lectin Fap2. Fap2 recognizes Gal-GalNAc, which is a sugar that is abundantly displayed by CRC cells^16^.

We hypothesized that, if oral *F. nucleatum* can translocate to colon tumors hematogenously, this bacterium may also reach Gal-GalNAc-displaying tumors in other organs via this route. To address this, we recently screened different cancer tissue samples for abundant Gal-GalNAc. This screen identified breast cancer as a strong candidate, which was in agreement with previous studies that had detected Gal-GalNAc in breast cancer^17–20^ or observed high accumulation of DNA from the genera *Fusobacterium* in the microbiome of malignant but not benign breast tissue samples^21^.

Breast cancer is the most commonly diagnosed cancer and the leading cause of cancer mortality in women^22^. Therefore, it is important to understand if *F. nucleatum* has the same profound impact on the progression and outcome of breast cancer as it has in CRC. Our present study provides several lines of molecular and experimental evidence for this to be the case. We demonstrate that Gal-GalNAc levels increase along the progression of human breast cancer and that *F. nucleatum* DNA is overabundant in human breast cancer samples, particularly in those with high Gal-GalNAc signals. Using two different murine orthotropic models, we show that *F. nucleatum* colonizes mammary tumors via abundant Gal-GalNAc on tumor cells, with the same Fap2 lectin whereby recognizes CRC cells. Furthermore, we find that the colonization of mammary tumors by *F. nucleatum* accelerates breast cancer progression and metastatic development, most likely through suppression of T cell accumulation in the tumor microenvironment. Importantly, breast tumor exacerbation by *F. nucleatum* in mice can be counteracted by antibiotic treatment with metronidazole.

## Results

### Gal-GalNAc is overdisplayed in breast cancer

**Fig. 1:**
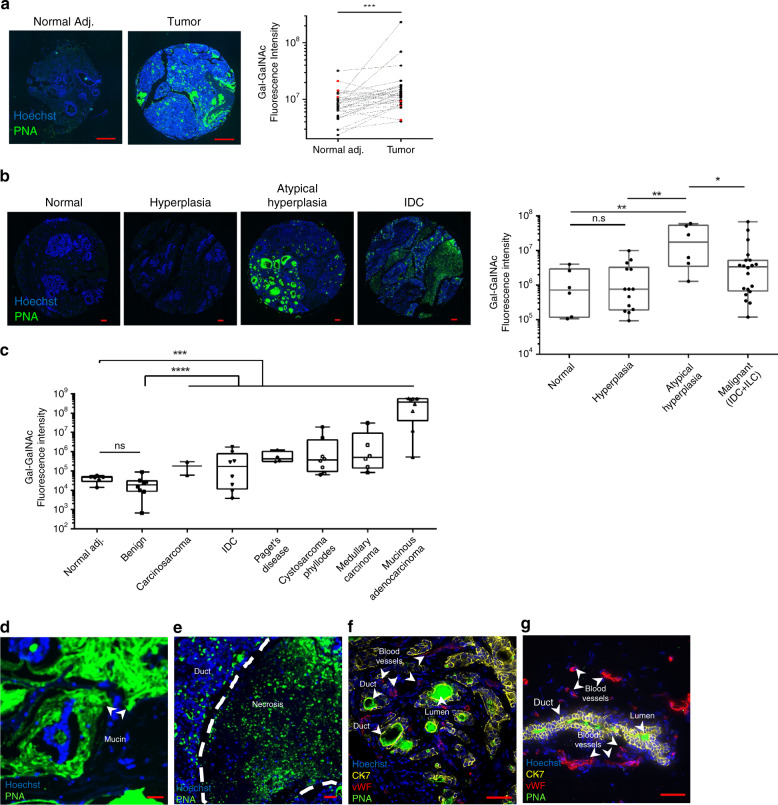
Gal-GalNAc is overdisplayed in breast cancer. **a** Representative images of human breast cancer and matched adjacent non-tumor (normal adj.) tissue using tissue microarray (HBre-Duc060CS-01) stained with FITC-labeled Gal-GalNAc-specific PNA lectin (green) and Hoechst dye (blue). Right panel: quantitative analysis of PNA binding (sum of fluorescence intensity) to each matching tumor and normal adjacent tissue cores. Each paired symbols represents one patient. Red symbols mark the five paired samples (out of 30) in which Gal-GalNAc level in the normal tissue was higher than that in the adjacent-tumor one. ***p = 0.0003, one-tailed, Wilcoxon matched-paired signed rank test, *n* = 30. **b** Representative images of human breast cancer hyperplasia, atypical hyperplasia, and normal tissue using tissue microarray (BR1003a) stained with FITC-labeled Gal-GalNAc-specific PNA lectin (green) and Hoechst dye (blue) (left panel). Right panel: quantitative analysis of PNA binding (sum of fluorescence intensity) to each tissue core. Each symbol represents one patient. **p = 0.0035 (normal–atypical hyperplasia), **p = 0.002 (hyperplasia–atypical hyperplasia), *p = 0.0415, n.s.: not significant, two-tailed Kruskal–Wallis test, normal *n* = 6, hyperplasia *n* = 14, atypical hyperplasia *n* = 6, malignant *n* = 20 (IDC + ILC, IDC: invasive ductal carcinoma, ILC: invasive lobular carcinoma). **c** Quantitative analysis of PNA binding (sum of fluorescence intensity) to types of human breast cancer, benign and normal adjacent (none matched) tissue using tissue microarray (BR1006) stained with FITC-labeled PNA. Each symbol represents one patient. ***p = 0.0004, ****p < 0.0001 between all the breast cancers and normal adj. and benign correspondingly, Holm-corrected Mann–Whitney test. *n* = 6 (normal adj.), *n* = 7 (benign), *n* = 36 (malignant). **d**–**g** Human breast cancer stained with FITC-labeled PNA (green), Cy5-anti-Cytokeratin-7 for glandular epithelia (yellow), TRITC-von willebrand factor for endothelial cells (red), and Hoechst dye (blue). High Gal-GalNAc levels can be detected in mucin regions in mucinous breast carcinoma (**d**), and in necrotic regions (**e**), and in duct and in duct lumen (**e**, **f**, **g**) of ductal breast cancer as indicated. Scale bar, 100 µm in (**a**, **b**), 50 µm in (**d**–**g**). Results presented in (**d**) and in (**e**–**g**) represent 2 and 3 reproducible independent experiments, respectively. Source data are provided as a Source data file.

We have shown previously that CRC-specific attachment and colonization by *F. nucleatum* is facilitated by high Gal-GalNAc levels on CRC cells, and that the Fap2-mediated recognition of this sugar is inhibited with soluble GalNAc or removal of the cell-exposed sugar with O-glycanase^16^. In addition, Fap2-deficient *F. nucleatum* showed impaired attachment to CRC samples, and much reduced CRC colonization in a mouse model^16^. Since Gal-GalNAc (also known as the Thomsen–Friedenreich antigen) was known to be displayed in breast cancer^17–20^, we hypothesized that *F. nucleatum* would target breast cancer tissue in a similar manner. To this end, we stained tissue microarrays containing human breast cancer and matched adjacent non-tumor tissue samples using fluorescein isothiocyanate (FITC)-labeled peanut agglutinin (PNA), a Gal-GalNAc specific lectin. In 25 of 30 tested paired samples, Gal-GalNAc levels in the tumor samples were higher than those in the matched normal tissue (Fig. 1a). In addition, breast cancer samples showed significantly higher (*p* = 0.0003) Gal-GalNAc levels than the matched adjacent non-tumor tissue (Fig. 1a).

We then compared Gal-GalNAc levels in samples from normal human breast tissue, breast tumor tissue including benign tumors, and from different stages along the progression of the disease. Gal-GalNAc signals were lowest in normal [median florescence intensity (MFI) 7.12 × 10^5^] and hyperplasia tissues (MFI 7.55 × 10^5^). Atypical hyperplasia (MFI 1.73 × 10^7^) and malignant (MFI 3.50 × 10^6^) tissue displayed 24- and 4.7-fold, respectively, higher levels of Gal-GalNAc than did normal tissue (Fig. 1b). Note that the high median of Gal-GalNAc levels in atypical hyperplasia might be also due to the low number of available samples. Interestingly, benign tumors (MFI 1.89 × 10^4^) showed significantly lower Gal-GalNAc levels than did the different malignant tumors (all with an MFI higher than 1.68 × 10^5^), and overall did not significantly differ from normal tissue (MFI 4.7 × 10^4^) (Fig. 1c). Comparing by subgroups of breast cancer, mucinous adenocarcinoma displayed at least two orders of magnitude higher amounts of Gal-GalNAc over the other five cancer subgroups tested (Fig. 1c). Of the latter, mucinous breast cancer showed the highest Gal-GalNAc levels in the mucin layers, whereas in ductal breast cancer high Gal-GalNAc levels were observed in necrotic regions, ducts, and in the ductal lumen (Fig. 1d–g).

### *F. nucleatum* gDNA is overabundant in breast cancer

Next, we tested whether *F. nucleatum* occurred in breast cancer, and if so, whether its presence correlates with high Gal-GalNAc levels. For this purpose, we searched for *F. nucleatum*-specific sequences in 50 deep sequencing libraries of PCR-amplified bacterial 16S rDNA from different breast cancer samples. Since breast tumors have low bacterial biomass, we included as negative controls for possible signals from contaminating DNA, 50 empty DNA extractions (controls) and 50 no-template PCR controls (NTCs). DNA from 21 CRC samples was included as positive controls.

**Fig. 2:**
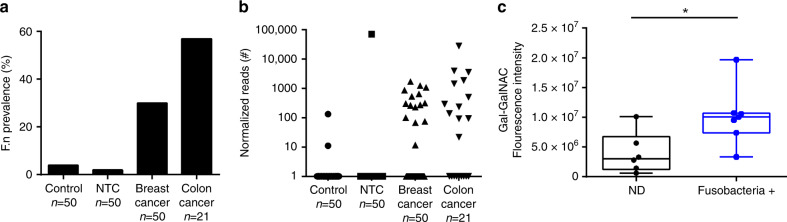
*F. nucleatum* gDNA is overabundant in breast cancer displaying high Gal-GalNAc levels. **a** The prevalence of *F. nucleatum* in breast tumors (*n* = 50) is shown and compared with DNA extraction controls (control, *n* = 50), PCR controls (NTC, *n* = 50), and colon tumors (*n* = 21). **b** Normalized read count (reads normalized to the average number of reads across different libraries) of *F. nucleatum* in each of the above samples (each dot represents the read counts of *F. nucleatum* in the sample) is shown (control *n* = 50, NTC *n* = 50, breast cancer *n* = 50, colon cancer *n* = 21). **c** Gal-GalNAc levels (measured using FITC-labeled PNA) in *F. nucleatum*-containing breast cancer samples (Fusobacteria+, *n* = 7) and in the samples in which *F. nucleatum* gDNA was not detected (ND, *n* = 6). *p = 0.0221, two-tailed Mann–Whitney test. Source data are provided as a Source data file.

We detected *F. nucleatum* in 30% and 57% of the analyzed breast tumors and CRC samples, respectively (Fig. 2a, b). Importantly, *F. nucleatum* was predominantly found in those breast cancer samples that displayed high Gal-GalNAc levels (Fig. 2c). These results not only confirm the previously observed prevalence of the *Fusobacterium* genera in the human breast cancer microbiome^21^, they also indicate a significant role of Fap2-Gal-GalNAc interactions in fusobacterial colonization of breast cancer tissue.

### Fap2 binds through Gal-GalNAc displayed on breast cancer

**Fig. 3:**
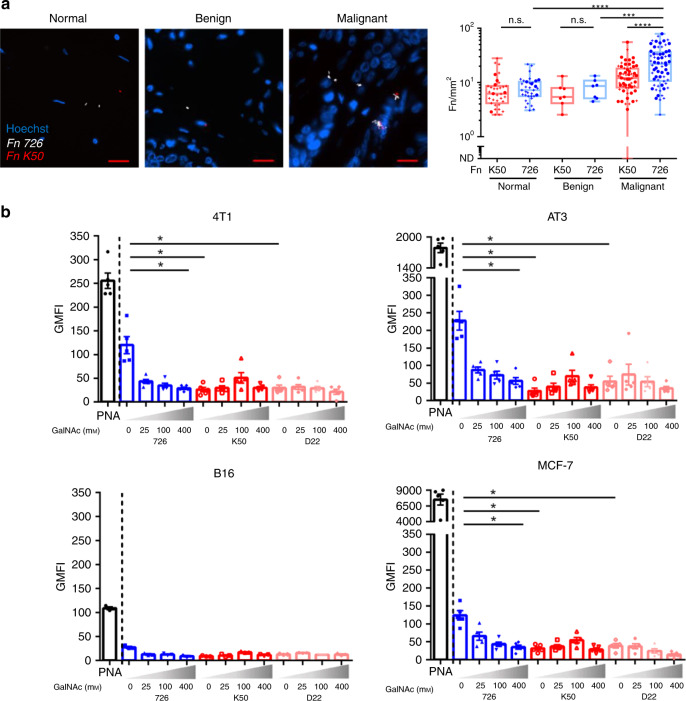
Attachment of *F. nucleatum* ATCC 23726 to breast cancer is Fap2-mediated and Gal-GalNAc dependent. **a** Representative images (left panels, scale bar 20 µm) and quantitative analysis (Fn/mm^2^) (right panel) of binding of Cy3-labeled (white) WT *F. nucleatum* ATCC 23726 (726) and of its Cy5-labeled (red) Fap2-inactivated isogenic mutant K50, to Hoechst-stained (blue) human breast cancer TMAs (HBre-Duc060CS-01 and BR1006). Results in the right panel are classified by tissue type (normal *n* = 35, benign *n* = 7, malignant *n* = 64) and bacterial strain (K50, 726). Each core is measured twice, once for each of the strains in the appropriate tissue type (as demonstrated in the pictures). When an individual contributed a single core to the experiment, the two observations are shown as circles (●); when she contributed two cores, these were to the normal type and the malignant type, and the four observations are depicted as plus signs (+). The six classes were compared against each other in pairs: the exact two-tailed Wilcoxon test was used for pairs that were from the same core, the exact two-tailed Mann–Whitney test for independent samples. Where the same hypothesis was tested on more than one pair, and the individual pairs were mutually independent, the corresponding *p*-values were combined by Fisher’s method. Wherever the pairs were not independent, the multiple comparisons were adjusted for using the Holm modification of the Bonferroni correction. ****p < 0.0001, ***p = 0.00079, n.s.: not significant. **b** Flow cytometry analysis of attachment of FITC-labeled PNA and of FITC-labeled *F. nucleatum* ATCC 23726 (726) and its Fap2-inactivated isogenic mutants K50 and D22 to breast cancer cell lines 4T1, AT3 (mouse cell lines), MCF-7 (human cell line) (*n* = 5 each), and mouse melanoma cell line B16 used as a control (*n* = 3), in the absence or presence of increasing amounts of soluble GalNAc. Bars indicate mean ± SEM. Each symbol represents average of 10,000 cells examined in duplicate. Data shown are representative of two independent experiments. *p = 0.0313, one-tailed, Wilcoxon matched-paired signed rank test (726, 0 mM–726, 400 mM GalNAc). *p = 0.0119 (all others), Holm-corrected one-tailed Mann–Whitney test. Source data are provided as a Source data file.

In our previous work establishing the Fap2 outer membrane protein as a major bacterial Gal-GalNAc lectin, we generated two Fap2-deficient mutants, K50 and D22, of *F. nucleatum* ATCC 23726^16,23^. Here, we used these strains to assay whether *F. nucleatum* through Fap2 specifically attaches also to breast cancer cells. Using microarrays containing samples of malignant and non-cancer (normal and benign) human breast tissue, we found that the Fap2-proficient wild-type strain (WT) ATCC 23726 exhibited significantly higher attachment to malignant breast cancer sections (median 21.96 Fn/mm^2^, Fig. 3a) than to normal (median 7.26 Fn/mm^2^) or benign breast tumor (median 8.59 Fn/mm^2^) sections. By contrast, the Fap2-deficient K50 mutant showed significantly impaired attachment to malignant breast cancer tissue (median 12.03 Fn/mm^2^), compared with the wild-type parental strain (median 21.96 Fn/mm^2^), while its attachment to normal breast sections (median 6.20 Fn/mm^2^) or benign tissue (median 5.41 Fn/mm^2^) was similar to WT (Fig. 3a). Of note, while the Fap2-deficient K50 mutant still attached better to breast cancer tissue, as compared with normal or benign sections, this tumor-specific attachment was not statistically significant. We speculate that other *F. nucleatum* tumor-binding factors such as FadA^3^, or the CEACAM1-binding CbpF outer-surface protein^24^ may enable *F. nucleatum* to recognize breast cancer cells even in the absence of Fap2.

Next, we used flow cytometry to compare attachment of WT versus Fap2-deficient *F. nucleatum* to several breast cancer cell lines, i.e., murine AT3 and 4T1, and human MCF-7. Mouse melanoma cell line, B16, served as negative control. Based on PNA binding, all three tested breast cancer cell lines displayed higher Gal-GalNAc levels than the B16 melanoma cell line (Fig. 3b, Supplementary Fig. 4). Similarly, *F. nucleatum* was found to attach only to the breast cancer cell lines. Supporting the predicted Fap2-mediated recognition of Gal-GalNAc, wild-type *F. nucleatum* ATCC 23726 showed significantly higher attachment than the K50 and D22 mutants (Fig. 3b). Addition of soluble GalNAc suppressed attachment, most likely by saturation of the Fap2 lectin (Fig. 3b). Interestingly, both wild-type and Fap2-deficient bacteria invariably attached better to AT3 cells than to MCF-7 or 4T1 cells, suggesting that the AT3 cell line possesses additional (surface) factors for fusobacterial attachment. In conclusion, the above results strongly argue *F. nucleatum* primarily uses its Fap2 lectin to home to breast cancer tissue with high levels of Gal-GalNAc.

### *F. nucleatum* preferentially colonizes breast cancer

Transient bacteremia is common in periodontal disease^13,25,26^, and provides an opportunity for oral fusobacteria to enter the circulatory system not only during dental treatment but also during daily routine. Once in the bloodstream, previously oral *F. nucleatum* might translocate to breast cancers hematogenously. To test this, we simulated transient bacteremia by intravascular injection of *F. nucleatum* in the 4T1 orthotropic BALB/c mouse model of mammary cancer. In this model, 4T1 cells are transplanted into the mammary fat pad of female BALB/c mice where they are highly tumorigenic and invasive.

**Fig. 4:**
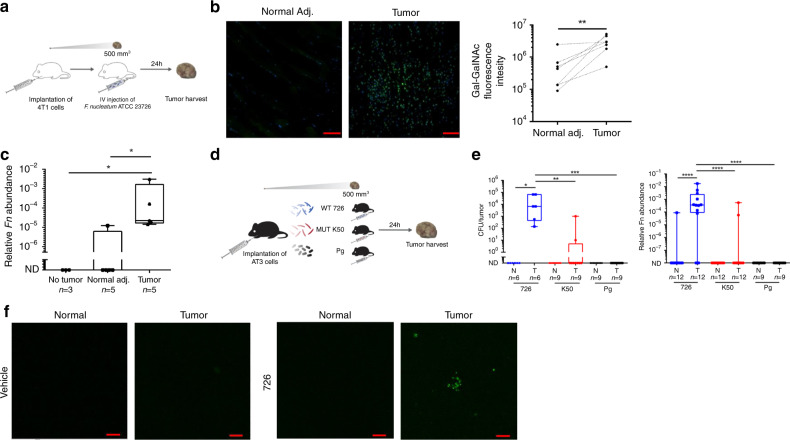
*F. nucleatum* colonizes mammary cancer in a Fap2-mediated mechanism. **a** Orthotropic 4T1 BALB/c mouse mammary cancer model. When reaching 500 mm^3^ size, 5 × 10^7^ *F. nucleatum* ATCC 23726 were IV injected. Twenty-four hours post inoculation, breast cancer and normal tissue from an adjacent mammary were harvested. **b** Representative images and sum of fluorescence intensity of binding of FITC-labeled PNA (green) to tumor and normal tissue stained with Hoechst dye (blue). Each paired symbols represents one mouse (*n* = 7), **p = 0.0078, one-tailed, Wilcoxon matched-paired signed rank test. **c** Relative *F. nucleatum* gDNA abundance (2^−ΔCt^) in breast tissue from tumor-free mice (No tumor, *n* = 3), and in tumor and matching normal tissue of 4T1 tumor-bearing mice (*n* = 5) all inoculated with *F. nucleatum* ATCC 23726. *p = 0.0358 Holm-corrected one-tailed Mann–Whitney test. *p = 0.0313, one-tailed, Wilcoxon matched-paired signed rank test (for paired normal adjacent-tumor samples). **d** Orthotropic AT3 C57BL/6 mouse mammary cancer model. When reaching 500 mm^3^, mice were inoculated with 5 × 10^7^ *F. nucleatum* ATCC 23726 (726; blue), 5 × 10^7^ Fap2 mutant K50 (MUT K50; red) or with 5 × 10^7^ *P. gingivalis* (Pg; black). Twenty-four hours later, normal mammary and breast cancer tissue were harvested from each mice and bacteria quantified. **e** Bacterial abundance (CFU/g tissue), and relative bacterial gDNA abundance (2^−ΔCt^) in tumor and normal tissue of AT3 tumor-bearing mice inoculated with *P. gingivalis* (*Pg*, *n* = 9), K50 (*n* = 12), or *F. nucleatum* ATCC 23726 (726, *n* = 12). Each symbol represents one mouse. *p = 0.0156 one-tailed, Wilcoxon matched-paired signed rank test. ***p = 0.0006, **p = 0.001598. Holm-corrected one-tailed Mann–Whitney test (left panel). ****p < 0.0001. One-tailed, Wilcoxon matched-paired signed rank test. ****p < 0.0001 (726 – Pg). One-tailed Mann–Whitney test, Holm’s modification of Bonferroni correction (right panel). **f** Representative multiphoton microscopy images of tumor and of normal adjacent mammary samples harvested 24 h post-IV injection of AT3 cancer bearing mice with 5 × 10^7^ FITC-labeled *F. nucleatum* ATCC 23726 or with PBS vehicle. Scale bar, 50 µm in (**b**), 10 µm in (**f**). Data shown are representative of three independent reproducible experiments. Source data are provided as a Source data file.

As illustrated in Fig. 4a, we transplanted 1 × 10^6^ 4T1 cells into the mammary fat pad, and when tumors reached a size of 500 mm^3^, mice were inoculated once with 5 × 10^7^ *F. nucleatum* ATCC 23726 by tail vein injection. Analysis of tissue 24 h post inoculation revealed that, consistent with our human breast cancer samples (see above), Gal-GalNAc levels (measured using FITC-labeled PNA) in the mouse cancer sections were higher (6.8-fold when comparing the medians) than in sections of matching normal tissues (Fig. 4b, right panel). We observed an at least two orders of magnitude higher median abundance of the bacteria, using *F. nucleatum* gDNA as a proxy, in the tumor sections than in the matching normal tissue (Fig. 4c). By contrast, we failed to detect bacteria in the mammary of control tumor-free mice (No tumor, Fig. 4c) treated the same way, indicating that blood-borne *F. nucleatum* require the presence of a tumor to localize to breast tissue.

### Fap2 mediates breast cancer colonization by *F. nucleatum*

To validate the observed mammary cancer colonization by *F. nucleatum* in a different animal model and evaluate the role of Fap2 in the process, we used the AT3 orthotropic mammary cancer model in C57BL/6 mice. Mice with tumors were generated as described above for the 4T1 model, yet this time injected with either wild-type *F. nucleatum* ATCC 23726, Fap2-deficient *F. nucleatum* K50, or the Gram-negative, anaerobic periodontal bacterium *Porphyromonas gingivalis* (ATCC 33277) as a control. Note that the latter species has been reported to be overabundant in oral squamous cell carcinoma^27–30^. Tumors and unaffected tissue from an adjacent mammary were analyzed 24 h post inoculation (Fig. 4d). As in the BALB/c model above, *F. nucleatum* ATCC 23726 was enriched in the tumor compared with adjacent normal tissue, as determined by both, colony counting and qPCR (Fig. 4e, left and right panels respectively). Fap2 deficiency (mutant K50) resulted in an at least a three orders of magnitude drop in mammary tumor colonization, as compared with WT bacteria (Fig. 4e). Interestingly, these results closely match those obtained with a CRC mouse model^16^. They imply that while neoplastic tissue certainly plays a critical role in enriching *F. nucleatum* in tumors, breast cancer-specific enrichment by *F. nucleatum* is crucially driven by Fap2.

Also in agreement with CRC colonization by *F. nucleatum*^20^, breast tumors are not colonized by just any oral anaerobic bacterium associated with periodontitis. This is to say that in mice inoculated with *P. gingivalis*, bacterial numbers in tumors were below the detection limit of culturing (∼2 CFU/g tissue) or qPCR (Fig. 4e). Thus, *F. nucleatum* likely possess distinctive features for tumor colonization, such as Fap2 (the focus of this work), FadA^3^ or CbpF^24,31^.

Finally, in order to directly visualize fusobacterial breast cancer colonization, we tracked FITC-labeled *F. nucleatum* ATCC 23726 in animals by multiphoton microscopy. To this end, we inoculated mammary tumor-bearing C57BL/6 (AT3 orthotropic mammary cancer model, see above) with 5 × 10^7^ FITC-labeled bacteria or injected PBS (the vehicle used to inject the bacteria) as control. Twenty-four hours post inoculation, we detected signals of fusobacteria in all tumor samples, whereas there was no signal in any of the adjacent normal mammary samples, nor in samples harvested from the sham-inoculated mice (Fig. 4f).

### Antibiotics inhibit *F. nucleatum*-induced tumor exacerbation

**Fig. 5:**
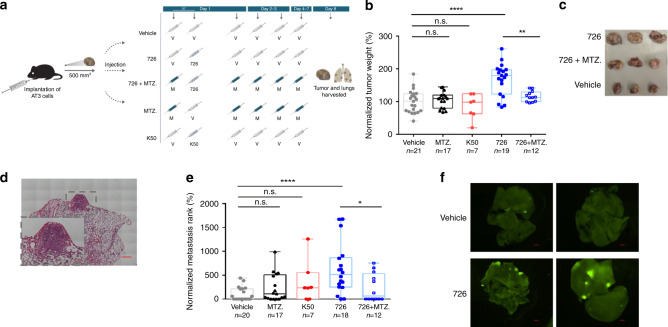
*F. nucleatum* ATCC 23726 accelerates tumor growth and progression of lung metastasis. **a** Experimental scheme: AT3 breast cancer cell line was injected to the mammary fat pad of 6–7-week-old C57BL/6 mice. When tumor size reached 500 mm^3^, PBS vehicle (V), 5 × 10^7^ *F. nucleatum* ATCC 23726 only (726), 5 × 10^7^ *F. nucleatum* ATCC 23726 with metronidazole (726+MTZ.), metronidazole only (MTZ.), or 5 × 10^7^ Fap2-deficient *F. nucleatum* K50 were injected into the tail vein. On days 2–3 post inoculation, mice were injected twice a day with metronidazole (M) or with PBS vehicle (V). On days 4–7 post inoculation, mice were injected once a day with metronidazole or with PBS. Eight days after inoculation, mice were sacrificed, and tumor and lungs harvested. Breast cancer tissues were weighed, and lung metastases were counted. **b** Tumor weights normalized as the percentage compared with the average tumor weight in the PBS group (vehicle control) in each experiment (set as 100% tumor weight). Each symbol represents one mouse. Presented results are of three independent experiments. ****p = 3.48 × 10^−5^, **p = 0.002. Holm’s correction, one-tailed Mann–Whitney. Vehicle *n* = 21, MTZ. *n* = 17, K50 *n* = 7, 726 *n* = 19, 726+MTZ. *n* = 12. **c** Representative tumors post-harvest. **d** Representative image of lung metastases. Scale bar, 500 µm in the large image, 250 µm in the indicated inset. **e** Lung metastasis rank [number of metastases per lung area (pixel^2^)] calculated as percentage compared with the average metastasis rank in the vehicle control group of each experiment (set as 100% metastasis rank). Each symbol represents one mouse. Presented results are of three independent experiments. ****p = 4.08 × 10^−5^, *p = 0.0177, n.s.: non-significant, Holm’s correction, one-tailed Mann–Whitney. Vehicle *n* = 20, MTZ. *n* = 17, K50 *n* = 7, 726 *n* = 18, 726+met *n* = 12. **f** Images taken by fluorescence binocular of lung metastases in four mice implanted with GFP-expressing AT3 cells as described above. Two tumor-bearing mice were inoculated with *F. nucleatum* and two were treated by PBS vehicle. Scale bar, 1000 µm. Source data are provided as a Source data file.

Given that colonization by *F. nucleatum* accelerates the formation of colonic tumor^3,4,11^, we used the AT3 orthotropic mammary cancer model to test whether it affects tumor progression in breast cancer as well, following the scheme in Fig. 5a. When tumors reached 500 mm^3^ in size (day 1), mice were randomly divided into five treatment (intravascular injection or inoculation) groups: PBS vehicle (V), wild-type *F. nucleatum* ATCC 23726 (726) or a Fap2 mutant (K50), *F. nucleatum* ATCC 23726 and additional treatment with metronidazole (726+MTZ.), or metronidazole alone (MTZ.). Starting with metronidazole (MTZ.) or PBS (V), bacteria were inoculated 30 min later. Treatment with metronidazole or PBS vehicle was repeated after 8 h, twice a day (morning and evening) in the following 2 days (days 2–3), and daily for 4 more days. On day 8, mice were sacrificed, and tumor and lungs were harvested to weigh breast cancer tissue and count lung metastases, respectively. As shown in Fig. 5b, mice inoculated with *F. nucleatum* ATCC 23726, but not with Fap2-deficient K50 bacteria, displayed significantly larger tumors than those in the control group. Metronidazole treatment alone did not affect tumor size (Fig. 5b), but did prevent tumor enlargement in mice inoculated with fusobacteria (Fig. 5b, c).

The median number of lung metastases in mice inoculated with *F. nucleatum* ATCC 23726 was at least two orders of magnitude higher than in the sham-inoculated mice (Fig. 5e). The *F. nucleatum*-driven increase in metastases numbers and size was also readily visible in mice bearing GFP-expressing AT3 tumors (Fig. 5f). By contrast, Fap2-deficient bacteria (K50), while promoting some increase in the number of metastases, did not cause a statistical significant higher burden of metastases. As with tumor size, metronidazole treatment prevented the pro-metastasis effect of *F. nucleatum* (Fig. 5e).

### *F. nucleatum* modulates immunity against mammary tumors

We considered several different mechanisms whereby *F. nucleatum* induces an increase in breast tumor size: (i) enhancing proliferation of the cancer cells; (ii) inhibiting apoptosis of the cancer cells; or (iii) manipulating anti-tumor immunity. Immunohistochemistry staining for the Ki67 proliferation marker or the apoptosis marker CC3 cleaved caspase 3 revealed no differences between *F. nucleatum*-infected and sham-infected tumors (Supplementary Fig. 1). Similarly, we found no indication for increased proliferation or reduced apoptosis in an RNA-seq based comparison of AT3 cells incubated for 24 h with or without *F. nucleatum* ATCC 23726 (Supplementary Fig. 3, Supplementary Data 1 and 2). These results suggested that breast tumor acceleration by *F. nucleatum* in the AT3 mouse model might be immune-mediated.

**Fig. 6:**
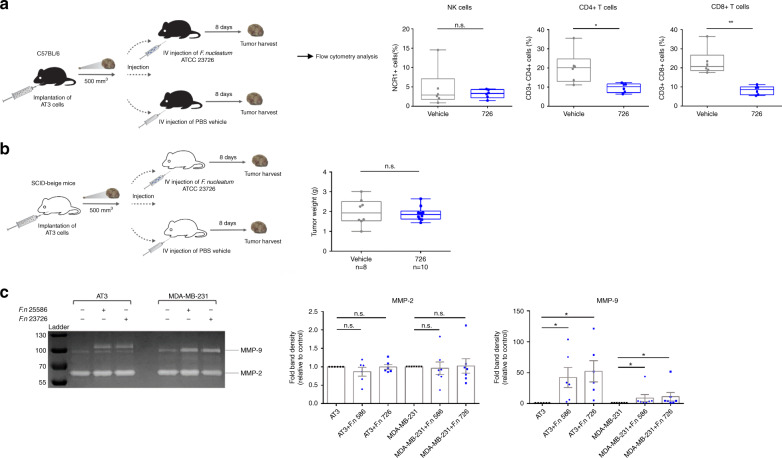
AT3 mammary tumor acceleration by *F. nucleatum* is immune-mediated. **a** 1 × 10^6^ AT3 cells were injected to the mammary fat pad of 6–7-week-old female C57BL/6 mice. When tumor volume reached 500 mm^3^, mice were IV injected with PBS vehicle or with 5 × 10^7^ *F. nucleatum* ATCC 23726 (726). At day 8 post injection, mice were sacrificed, tumors were harvested subjected to flow cytometry and abundance of NK cells, CD4+ T cells and CD8+ T cells was determined. Boxplot whiskers represent extrema, box bounds represent upper and lower quartiles, and center-line represents the median value. n.s.: non-significant. **p = 0.00216, *p = 0.0152, two-tailed Mann–Whitney (each symbol represents the mean of two technical replicates, *n* = 6 per group). **b** 1 × 10^5^ AT3 or GFP-expressing AT3 cells were injected to the mammary fat pad of 6–7-week-old female SCID-beige mice. When tumor volume reached 500 mm^3^, mice were IV injected with PBS vehicle or with 5 × 10^7^ *F. nucleatum* ATCC 23726 (726). At day 8 post injection, mice were sacrificed, tumors were harvested and weighed in an analytic weigh. Boxplot whiskers represent extrema, box bounds represent upper and lower quartiles, and center-line represents the median value of two independent experiments. n.s.: non-significant. *n* = 8 per vehicle group and *n* = 10 per *F.* *nucleatum*-infected group. **c** Representative picture and densitometry analysis of gelatin zymography of conditioned medium prepared from mouse mammary tumor cell line AT3 or from the human breast cancer cell line MDA-MB-231 that were co-cultured or not, with *F. nucleatum* strains ATCC 25586 or ATCC 23726. Bars indicate mean ± SEM of six independent experiments. *p = 0.031 two-tailed paired Wilcoxon. Note that mouse MMP-9 is larger than that of human. Source data are provided as a Source data file.

We previously discovered that *F. nucleatum* can inhibit anti-tumor immunity by activating two human (though not the mouse homologs of) immune-suppression checkpoint receptors, TIGIT and CEACAM1^8,31^. In addition, tissue load of *F. nucleatum* was previously found to be inversely correlated with density of tumor-infiltrating lymphocytes in colorectal carcinoma tumors^5–7^. Therefore, to determine whether immune cell accumulation might be affected by *F. nucleatum*, we used flow cytometry to compare levels of NK cells, CD4+ T cells and CD8+ T cells between AT3 mammary tumors from *F. nucleatum*-infected and uninfected mice. This analysis showed that inoculation by *F. nucleatum* resulted in fewer CD4+ and CD8+ T cells (Fig. 6a, Supplementary Fig. 5a–c).

To evaluate the involvement of T, B, and NK cells in fusobacterial acceleration of the progression of the AT3 tumor, we repeated our *F. nucleatum* inoculation scheme in SCID-beige mice, which lack T, B, and NK cells. Unlike in C57BL/6 mice, AT3 tumors grown in uninfected SCID-beige mice were not smaller than those in the infected mice (Fig. 6b). We interpret this finding to suggest that in the immune-competent C57BL/6 mice, growth of AT3 breast tumor is restricted by T, B, or NK cells, and that *F. nucleatum* directly or indirectly dampens this immune-mediated tumor control.

Searching the above-mentioned RNA-seq data for additional potential mechanisms whereby the presence of *F. nucleatum* might accelerate development of breast tumors, we noticed an overexpression of matrix metalloproteinase 9 in the AT3 cells incubated with *F. nucleatum* (Supplementary Fig. 3). Proteases of the matrix metalloproteinase (MMPs) family play vital roles in many biological processes that involve matrix remodeling. In particular, MMP-9 activity has been related to cancer pathology including invasion, angiogenesis, and metastasis^32^. While many MMPs expressed in breast cancer are produced by stromal cells, MMP-9 is produced mainly by the tumor cells themselves^32^. Echoing this general patterns, a gel zymography assay with gelatin as substrate showed unaltered secretion of MMP-2 by cell lines AT3 (murine mammary tumor) or MDA-MB-231 (human breast cancer) incubated with *F. nucleatum* ATCC 23726 or with *F. nucleatum* ATCC 25586; by contrast, the secretion of MMP-9 was significantly and invariably increased when both cell lines where incubated with each of the two *F. nucleatum* strains (Fig. 6c). Therefore, in addition to immune modulation as the putative major mechanism of *F. nucleatum* in the AT3 mouse model in C57BL/6 mice, induction of MMP might be another mechanism whereby *F. nucleatum* accelerates breast tumor progression.

## Discussion

The involvement of viruses in tumor development has been investigated for over a century, whereas the interest in potentially similar roles of bacteria in cancer biology has been rather recent, with the primary focus being the carcinogenic role of *Helicobacter pylori* in gastric cancer^33,34^. Periodontitis is a common bacterial-induced inflammatory disease. Existing data provide support for positive association between periodontitis and risk of cancer^35,36^, particularly oral, lung, and pancreatic cancers^37^. A recent meta-analysis also predicted that periodontal disease increased the risk of breast cancer by 1.22-fold^38^.

Of the many different bacteria presently linked to cancer development, *F. nucleatum* has so far been associated only with colorectal and esophageal cancers. Being an oral microbe, it was plausible that *F. nucleatum* reaches CRC tissue by descending through the digestive tract^4^. However, our recent finding that oral *F. nucleatum* might probably colonize CRC via the hematogenous route, and its selectivity for tumors that display Gal-GalNAc^16,20^, suggested that additional tumors might be colonized by this bacterium. As to a specific candidate cancer, we have demonstrated here that Gal-GalNAc levels increase as breast cancer progresses (Fig. 1a, b), supporting earlier reports of Gal-GalNAc detection in breast cancer samples^17–20^.

Breast cancer represents a sequence of events, starting with non-neoplastic epithelium and subsequent progress through the stages of hyperplasia, atypical hyperplasia, carcinoma in situ and invasive adenocarcinoma. It has been hypothesized that the conversion between benign hyperplasia and carcinoma in situ (the stage preceding invasive carcinoma) is made at the transition from hyperplasia to atypical ductal hyperplasia^39^. Interestingly, we observed the sharpest rise in Gal-GalNAc levels in this very transition phase (Fig. 1b). In addition, Gal-GalNAc levels in breast cancer were 4.7-fold higher than in normal tissue (Fig. 1b), but only 1.4-fold higher than in matched normal adjacent tissue (Fig. 1a). These results suggest that the proximal tumor also affects the adjacent non-tumor tissue, causing aberrant Gal-GalNAc levels in the latter. These results are in line with previous ones demonstrating that normal tumor adjacent tissue represents an intermediate state between healthy and tumor^40^.

The sum of our in vitro and in vivo results strongly support a model whereby *F. nucleatum* colonizes not only colorectal cancer^16^, but also breast cancer through recognition of Gal-GalNAc by Fap2. They also imply, that as in CRC^3,4,16^, colonization of breast cancer by *F. nucleatum* is secondary to tumor initiation: in the absence of tumors, no *F. nucleatum* bacteria were detectable in the mouse mammary glands. Colonization likely occurs as blood supply is attracted to the tumor, enabling sufficient bacterial trafficking from the mouth, and after tumor Gal-GalNAc levels raised. Importantly and in agreement with its role in CRC^3,4^, colonization by *F. nucleatum* accelerated the progression of mammary tumor, yet strictly in a Fap2-dependent manner (Fig. 5b, c). That is, tumors were not enlarged in mice inoculated with Fap2-deficient bacteria.

Since *F. nucleatum* only activates human TIGIT and CEACAM1 and not their murine homologs^8,31^, tumor acceleration in the mouse models used here cannot involve impaired T cell and NK cell killing-activity via the activation of these important checkpoints. Nonetheless, our observation of reduced CD4+ and CD8+ T cell levels in *F.* *nucleatum*-infected tumors (Fig. 6a), and the lack of acceleration of AT3 tumors by *F. nucleatum* in SCID-beige mice (Fig. 6b), both indicate a role of immunity in fusobacterial tumor acceleration in the AT3 mouse model. Thus, it appears that in the immune-competent C57BL/6 mice, growth of AT3 breast tumor is normally restricted by NK, B, or T cells, but in the presence of *F. nucleatum* T cells become reduced, resulting in tumor growth acceleration. The reduction of immune cells might involve apoptosis. Apoptosis was shown to be induced by *F. nucleatum* in lymphocytes, through its lectin Fap2^41^. Importantly, we expect the pro-tumorigenic effect of *F. nucleatum* to be much stronger in humans because the activity of the NK and few T cells present in the tumor will be further weakened by inhibitory interactions of Fap2 with TIGIT and of CbpF with CEACAM1.

There are several important similarities of our results with previous observations with CRC. First, lower numbers of T cells have also been reported in CRC samples containing *F. nucleatum*^5–7^. Second, metronidazole treatment to target *F. nucleatum* reduces tumor growth not only in CRC^11^, but also prevents fusobacteria-associated acceleration of breast cancer (Fig. 5b, c). Since the metronidazole treatment had no effect on tumor development in the absence of *F. nucleatum*, it must be the tissue sterilization by the antibiotic that nullifies the fusobacterial-mediated tumor acceleration (Fig. 5b).

Metastasis causes as much as 90% of cancer-associated mortality in general, and is also the main cause of death by breast cancer. Whereas patients with localized breast cancer have a 5-year survival rate of 98%, this drops to 26% in patients with metastatic breast cancer^42^. In our mouse model, we observed not only an enlargement of primary AT3 mammary tumors by *F. nucleatum*, but also a significant increase in the number of lung metastases (Fig. 5e, f). The fusobacterial-driven increase in metastasis was clearly dependent on Fap2, i.e., it was not statistically significant with the Fap2-deficient K50 mutant (Fig. 5e).

Further work is needed to delineate the pro-metastasis mechanisms of *F. nucleatum*, for example, whether the increase in metastasis load is due to *F. nucleatum* increasing the size of the primary tumors. However, if we normalize metastases number by tumor weight, we see a higher metastasis burden in the lungs of mice with *F. nucleatum* than in those without bacterial inoculation (Supplementary Fig. 2). In other words, there are more lung metastases in the presence of *F. nucleatum*, even if the primary tumors are of the same size as in non-inoculated mice. In addition, 8 days post *F. nucleatum* infection may be too short to detect new metastasis with H&E staining used here. We therefore conclude for now that it was the *F. nucleatum*-driven growth acceleration of pre-infection metastases that led us to observe more metastases at the end of the experiment. This hypothesis is also supported by the visualization of lungs metastases in mice implanted with GFP-expressing AT3 cells, where the metastases in mice infected with *F. nucleatum* are clearly larger than those in the sham-infected mice (Fig. 5f).

Finally, our current study supports the previous important observation of overabundant DNA of the genus *Fusobacterium* in the human breast cancer microbiome^21^. Moreover, we show here that *F. nucleatum* DNA signals in human breast cancer correlate with the extent of Gal-GalNAc displayed by the tumors (Fig. 2c). Together, these results provide strong evidence for a model whereby *F. nucleatum* generally reaches tumors via the hematogenous route and specifically attaches to them via a bacterial lectin-host sugar (Fap2-Gal-GalNAc) interaction. This new knowledge, combined with our demonstration that metronidazole treatment counteracted acceleration of development and metastatic progression in mammary tumors (Fig. 5), suggests that targeting *F. nucleatum* might benefit treatment not only of CRC^11^ but also of breast cancer.

## Methods

### Human sample acquisition and DNA extraction

Tumor samples were collected and analyzed according to IRB-approved protocols by the Sheba Medical Center and the Maccabi Health Care Services. Fifty formalin-fixed paraffin-embedded (FFPE) samples from breast tumors and 21 fresh frozen colon tumor samples were collected after informed consent was obtained. DNA was extracted with the UltraClean Tissue & Cells DNA Isolation kit (MoBio) according to the manufacturer’s instructions with a 10 min bead beating step (Vortex adapter –Qiagen) before proteinase K digestion. Extraction of DNA from FFPE samples was done as described above with some adaptations. Briefly: samples were deparaffinized prior to digestion with proteinase K. Lysates were bead beated with 0.1 mm zirconia/silica beads (Biospec) for 10 min. Fifty empty tubes were added to the DNA extraction and were subjected to 16S sequencing together with sample DNA in order to detect and monitor contaminating bacterial DNA.

### 16S sequencing and data analysis

16S-rDNA amplification and sequencing were performed using a novel method that allows the processing of DNA from FFPE samples and increases the resolution of the assay because it amplifies five regions on the 16S rRNA gene. The method is described in detail in ref. ^43^. Fifty no-template controls (NTC) were added to the PCR amplification and further processed into libraries to differentiate (together with the extraction controls) contaminating bacterial DNA from true signal in the samples. Five regions of the 16S rRNA gene were amplified using 100 ng DNA as an input and a set of 10 multiplexed primers (Supplementary Table 1). Sequencing libraries were loaded together with 20% of PhiX on an Illumina Hi-seq or Mi-seq system. Reads were demultiplexed per sample, filtered and aligned to each of the five amplified regions based on the primers’ sequences. The SMURF package was applied to combine read counts from the five regions into a coherent profiling results solving a maximum likelihood problem^44^. The Greengenes database (May 2013 version) was used as a reference.

### Bacterial strains and growth conditions

*F. nucleatum* strains ATCC 25586, ATCC 23726, the isogenic Fap2-inactivated mutants K50, D22 and *P. gingivalis* ATCC 33277 were grown in Wilkins Chalgren broth (Oxoid, UK) or on Columbia agar plates (Oxoid, UK) supplemented with 5% defibrinated sheep blood (Novamed, Israel). The bacteria were grown in an anaerobic chamber (Bactron I–II Shellab, USA) in an atmosphere of 90% N_2_, 5% CO_2_, and 5% H_2_ at 37 °C. Thiamphenicol (2.5 µg/ml) was added for K50 and D22.

### Tissue microarray analysis

Breast cancer tissue array BR1006, array BR1003a, and HBre-Duc060CS-01 (US Biomax) were used in these studies. Details about the cases for each core on the array are available on the US Biomax Web site (https://www.biomax.us/).

### Fluorescence microscopy and section preparation

TMA slides were stained with H&E or processed for immunofluorescence microscopy. TMA slides went through deparaffinization as follows: xylene twice 5 min, xylene 1 min, xylene/ethanol (50%/50%) 1 min, ethanol twice 10 min, 90% ethanol 2 min, 80% ethanol 2 min, 70% ethanol 2 min and then washed in DDW. Slides went under heat-induced epitope retrieval using sodium citrate 10 mM pH 6.0 in a microwave for 20 min, then 40 min in room temperature and washed 5 min in PBS. For PNA (Sigma-Aldrich), anti-CK-7 (Abcam) and anti-von willebrand factor (Dako) binding, sections were blocked with PBS supplemented with 10% BSA, 10% FBS, and 5% Triton for 2 h at room temperature followed by incubation with FITC-labeled PNA (50 µg/ml in PBS), anti-CK-7 (1:200), or anti-von willebrand factor (1:500) overnight at 4 °C. The slides were then washed three times with PBS for 15 min and for anti-CK-7 and anti-von willebrand factor a second antibody Cy5 and Cy3 was added, respectively (Jackson, 1:100), for 2 h, washed three times with PBS for 15 min and then incubated with Hoechst 33258 diluted 1:5000 for 15 min at room temperature. Fluorescence intensity FITC-labeled PNA was evaluated using the ImagePro Analyzer 7.0 software (Cybernetics, USA). For *F. nucleatum* binding, bacteria were labeled with Cy5 or Cy3 (PA25001 Life Sciences GE) solution diluted 0.1 mg/ml in PBS. Sections were blocked with TBS (0.05 M Tris-HCl [pH 7.8], 0.1 M NaCl) supplemented with 20% BSA, 20% FBS, and 5% Triton for 6 h at room temperature, followed by incubation with the labeled bacteria (3 × 10^7^ bacteria/ml blocking solution) for 6 h at room temperature and then overnight at 4 °C. The slides were then washed once with PBS + TWEEN 0.5% followed by two washes with PBS for 15 min each, and then incubated with Hoechst 33258 diluted 1:5000 for 15 min at room temperature.

### Immunohistochemistry

Two slides 10-µm thick and 10 µm apart were prepared from the center of each formalin-fixed paraffin-embedded tumor. Slides were stained with Hematoxylin and one for ki67 (ɑ ki67—ThermoScientific (SP6) RM—9106-SO, diluted x 200) and the other for CC3 (ɑ CC3—Cell Signaling—9661S, diluted x 300). 10 randomly areas from each slide were captured in ×20 lens. Binary area (µm^2^) of ten areas were averaged for both DAB (+) and Hematoxylin (+). Each symbol represents the ratio between the average of DAB (+) to Hematoxylin (+) in the tumor.

### Cell lines and tissue culture

The human breast cancer cell line MCF-7 and MDA-MB-231, mouse BALB/c breast cancer model cell line 4T1, mouse C57BL/6 breast cancer model cell line AT3, and mouse C57BL/6 melanoma model cell line B16, were cultured with DMEM with 10% fetal bovine serum, 1% L-Glutamine, penicillin–streptomycin and amino acid (Biological Industries).

### Flow cytometry

*F. nucleatum* strains ATCC 23726 and the isogenic Fap2-inactivated mutants K50, D22 (10^9^ CFU/ml) were labeled with fluorescein isothiocyanate (FITC, 0.1 mg/ml in PBS; Sigma-Aldrich) for 30 min at room temperature and washed three times in PBS. FITC-labeled bacteria were used at a multiplicity of infection of 3. Bacteria were incubated with cells in 96-well plates, for 30 min at room temperature and washed twice prior to flow cytometry (LSRFortessa Analyzer, BD, USA). Analysis was performed using FlowJo 10.0.8 software (Tree Star, Ashland, OR, USA). FITC-labeled PNA lectin (Sigma-Aldrich) was incubated at a final concentration of 140 nM per 2.5 × 10^5^ cells per well. For competition experiments, bacteria were incubated with GalNAc (concentration range: 0, 25, 100, and 400 mM) for 30 min prior to incubation with cells.

### Animal models

All mouse experiments were carried out under protocol MD-17-15239-5 approved by the Hebrew University of Jerusalem Ethics Committee and signed by the chairman Prof. Sara Eyal. Six- to seven-week-old female Balb/C, C57BL/c, and SCID-beige mice were purchased from Envigo (Israel). Mice were kept at a relative humidity 40–70%, 20–24 °C, and in 12 h dark/light cycles (07:00–19:00 light).

### In vivo breast cancer colonization

Female BALB/c mice were orthotopically (mammary fat pad) injected with 1 × 10^6^ 4T1 tumor cells. When tumor reached 500 mm^3^ size, mice were injected intravenously with 5 × 10^7^ *F. nucleatum* ATCC 23726. Female C57BL/c mice were orthotopically (mammary fat pad) injected with 1 × 10^6^ AT3 tumor cells. At tumor size of 500 mm^3^, mice were randomly divided to three groups and injected intravenously with 5 × 10^7^ *F. nucleatum* ATCC 23726, *F. nucleatum* MUT K50 and *P. gingivalis* ATCC 33277. After 24 h, breast tumor and normal tissue from an adjacent mammary were harvested from each mice and homogenized under sterile conditions.

### Quantification of bacteria using plating and qPCR

Tumor and non-tumor adjacent tissue samples were homogenized in 500 µl of PBS for 45 s at 4.5 m/s using a FastPrep (MP Biomedicals, USA) and plated on Columbia agar plates supplemented with 0.15% (final concentration) crystal violet and 5% (final concentration) defibrinated sheep blood^45^. Colonies were enumerated after 7 days of incubation under anaerobic conditions. DNA was extracted using the DNeasy Blood & Tissue Kit (Qiagen, Germany) according to manufacturer’s instructions. A custom TaqMan primer/probe set was used to amplify *F. nucleatum* and *P. gingivalis* DNA. The cycle threshold (Ct) values were normalized to the amount of murine gDNA in each reaction by using a primer/probe set for the reference gene (Gapdh). Each reaction contained 1 ng of DNA and was assayed in triplicate in 20 µL reactions containing 2× qPCRBIO Lo-ROX Probe Mix as appropriate for individual qPCR machines. Reaction conditions were as follows: 2 min at 50 °C, 10 min at 95 °C, and 40 cycles of 15 s at 95 °C, and 1 min at 60 °C^16^. The sequences of all primers can be found in Supplementary Table 1.

### Multiphoton microscopy of FITC-labeled *F. nucleatum*

AT3 breast cancer cell line was injected to the mammary fat pad of 6–7-week-old female C57BL/6 mice. At tumor size of 500 mm^3^, mice were randomly divided to two groups; one group injected intravenous with 5 × 10^7^ FITC-labeled *F. nucleatum* ATCC 23726 (*n* = 5) and the second control group, with PBS vehicle (*n* = 3). After 24 h, breast tumor tissue was harvested from each mice. A slice from the fresh tissue was cut and placed on a slide with a drop of water and covered with a cover slip. Image sections were taken using a Nikon Multiphoton A1MP set to 740 nm wavelength using ×25 objectives.

### Effect of *F. nucleatum* on the progression of mammary tumor

AT3 breast cancer cell line was injected to the mammary fat pad of 6–7-week-old female C57BL/6 mice. When tumor reached 500 mm^3^ size (day 1), mice were randomly divided into five IV injected treatment groups: PBS (V), 5 × 10^7^ *F. nucleatum* ATCC 23726 and treated with metronidazole (726+MTZ.), metronidazole only (MTZ.), 5 × 10^7^ *F. nucleatum* ATCC 23726 treated with PBS vehicle (726), or with 5 × 10^7^ Fap2-deficient *F. nucleatum* K50 treated with PBS (K50). For metronidazole or vehicle treatment, mice were injected with metronidazole or PBS 30 min before IV inoculation with *F. nucleatum*. Treatment with metronidazole or vehicle was repeated after 8 h, twice a day (morning and evening) in the following 2 days (days 2–3), and daily for 4 more days. On day 8 mice were sacrificed, and tumors and lungs were harvested. Breast cancer tissue was weighed and metastases were counted by histological examination (experimental scheme can be seen Fig. 4a). When the GFP-expressing AT3 breast cancer cell line was implanted in C57BL/6 mice, relatively few mice developed cancer presumably due to anti-GFP immunity. In mice in which breast tumor was developed, metastases were visualized using Nikon SMZ25 fluorescent binocular in an ×0.5 objectives.

### Characterization of AT3 tumor immune infiltrate

Lymphocytes were purified from homogenized primary tumors by centrifugation on Lymphoprep (STEMCELL Technologies, Cat number 07851). Lymphocytes were characterized using anti-mouse CD3-allophyco-cyanin clone 145-2C11 (Cat. number 100312, BioLegend) for T cells together with either anti-mouse CD4 PE clone GK1.5 (Cat. number 100408, BioLegend) or anti-mouse CD8 PE clone 53-6.7 (Cat. number 100708, BioLegend). For the characterization of NK cells we used anti-mouse NKp46 (CD335, NCR1) APC clone 29A1.4 (Cat. number 137608, BioLegend).

### RNA-Seq

Infection of AT3 cells was carried out as describe before^46^ with the following modifications. Two days prior infection 2 × 10^5^ AT3 cells were seeded in six-well plates and 2 ml complete DMEM. The plates were transferred to an incubator with O_2_-control set to 1% O_2_ and the media exchanged after 24 h with complete DMEM without antibiotics. A day before infection, *F. nucleatum* ATCC 23726 was inoculated in Columbia broth under anaerobic conditions. On the next day, the culture was diluted 1:50 in fresh Columbia broth and grown to an OD_600_ of 0.2. Bacterial cells were harvested (5 min at 4400 × *g* in room temperature) resuspended in fresh, reduced complete DMEM (lacking antibiotics) and added to the AT3 cells at a multiplicity of infection (m.o.i.) of 10. The plates were centrifuged for 5 min at 250 × *g* at room temperature and placed back into the O_2_-controlled incubator. Twenty-four hours after infection, the cells were washed once with PBS before extracting total RNA using the mirVana kit (Ambion) following the manufacturer’s instructions. The infection was performed in three biological replicates. cDNA libraries for Illumina sequencing were generated by the Core Unit SysMed (University Würzburg). Five hundred nanograms of total RNA were used for the library generation using oligo-dT capture beads for poly-A-mRNA enrichment via the TruSeq Stranded mRNA Library Preparation Kit (Illumina) according to manufacturer’s instructions. The success of the library preparation was controlled by electrophoresis on Agilent High Sensitivity Bioanalyzer microfluidic chips prior to sequencing of pooled libraries (including 1% PhiX control library). For sequencing the single-end mode on the NextSeq 500 platform (Illumina) with the High-Output Kit v2.5 (75 cycles) was used. The generated RNA-seq data was deposited in NCBI’s Gene Expression Omnibus and are accessible through GEO Series accession number GSE144143.

FASTQ files were generated and quality filtered using the local run manager software from Illumina (v2.2.0) and FastQC (v.0.11.7; https://www.bioinformatics.babraham.ac.uk/projects/fastqc/). Mapping was performed using the READemption^47^ tool (v0.4.3) using the *Mus muculus* reference sequence provided by ENSEMBL (GRCm38.p6).

After mapping, strand-specific gene-wise quantification was performed using READemption based upon the annotation provided by ENSEMBL (GRCm38.p6). Only uniquely mapped reads were used as input for differential expression analysis via the edgeR package (v3.24.3)^48,49^ using an upper-quartile normalization and a prior count of 1. Only gene showing at least 10 uniquely mapped reads were considered for the analysis and all genes with an adjusted *p-*value of 0.05 and passing the log_2_ fold change threshold (−2 ≤ log_2_FC ≥ 2) were considered significant differentially expressed.

In order to assess enrichment of genes involved in different processes a gene set enrichment analysis (GSEA^50,51^ v4.0.3) was performed using the curated Molecular Signatures Database (MSigDB 7.0) for GO terms (biological processes) available via GSEA. The log_2_ fold changes reported by the edgeR analysis were used as input for the GSEA which was run in ranked list mode (statistic setting: classic) and limited to gene sets with 15–500 genes. An overview of all GO terms reported with an FDR-corrected *p*-value ≤0.05 are reported in Supplementary Data 1 and the top 15 based upon the normalized enrichment score are shown in Supplementary Fig. 3.

### MMPs detection using gel zymograpy

AT3 and MDA-MB-231 cells were cultured on 6-well plate at 37 °C in DMEM medium supplemented with 10% serum and 1% L-glutamine for 24 h to reach subconfluent density under microaerobic conditions using the Oxoid CampyGen atmosphere generating system. *F. nucleatum* strains ATCC 25586 and ATCC 23726 were brought to OD_600_ = 1 and added to the cell cultures at cell to bacterial ratio of 1:100. The cultures were then grown for 24 h in the absence of serum. Growth media were collected and conditioned by passing through 0.22 µm filter to remove bacteria and cancer cells, and concentrated 10X using 50 kDa membrane filters (Amicon Ultra 50k). For zymogram analysis^52^, 20 µg of conditioned media were dissolved in sample buffer (192 mM Tris-HCl [pH 6.8], 30% glycerol, 9% SDS) without β-mercaptoethanol and without boiling, and subjected to SDS-PAGE using 7.5% gels containing 1 mg/ml Gelatin from porcine skin (Sigma-Aldrich, Germany). Following electrophoresis, the gels were washed twice for 30 min at room temperature with washing buffer (50 mM Tris-HCl [pH 8.0], 5 mM CaCl_2_, 1 µM ZnCl_2_, 0.016% NaN_3_), containing 2.5% Triton X-100, then incubated for 10 min in incubation buffer (washing buffer containing 1% Triton X-100) and incubated overnight at 37 °C with fresh incubation buffer. Proteolytic activity was visualized as a clear band against a blue background after staining with Coomassie brilliant blue R-250.

### Statistical analysis

The various groups were compared against each other in pairs: the exact Wilcoxon test was used for pairs that were matched, the exact Mann–Whitney test for independent samples (Nonparametric Tests, IBM SPSS Statistics for Windows, Version 25, 2017, Armonk, NY: IBM Corp.). When the same hypothesis was tested on more than one pair, and the individual pairs were mutually independent, the corresponding *p*-values were combined by Fisher’s method^53^. Wherever the pairs were not independent, the multiple comparisons were adjusted for using the Holm modification^54^ of the Bonferroni correction.

In all presented boxplots, whiskers represent extrema, box bounds represent upper and lower quartiles, and center-line represents the median value.

### Reporting summary

Further information on research design is available in the Nature Research Reporting Summary linked to this article.

## Supplementary information

Supplementary Information

Description of Additional Supplementary Files

Supplementary Data 1

Supplementary Data 2

Reporting Summary

## Data Availability

The raw 16S sequencing data have been deposited in the NCBI BioProject database- accession # PRJNA624822. The RNA-seq data that support the findings of this study has been deposited in GEO with the accession code GSE144143. The source data underlying Figs. 1a–c; 2a–c; 3a, b; 4b, c, e; 5b, e; 6a–c; Supplementary Figs. 1 and 2 are provided as a Source data file. Remaining data is available in the Article, Supplementary Information files or available from the authors upon request.

## Source data

Source Data
