# Supplementary material for: Breast cancer colonization by Fusobacterium nucleatum accelerates tumor growth and metastatic progression
Source: Nat Commun. 2020 Jun 26;11:3259. doi: 10.1038/s41467-020-16967-2 (PMC7320135; doi:10.1038/s41467-020-16967-2)
Supplement: Supplementary file 1 — Supplementary Information [file 41467_2020_16967_MOESM1_ESM.pdf]

Breast cancer colonization by *Fusobacterium nucleatum* accelerates tumor growth and metastatic progression

Parhi, Alon et al.

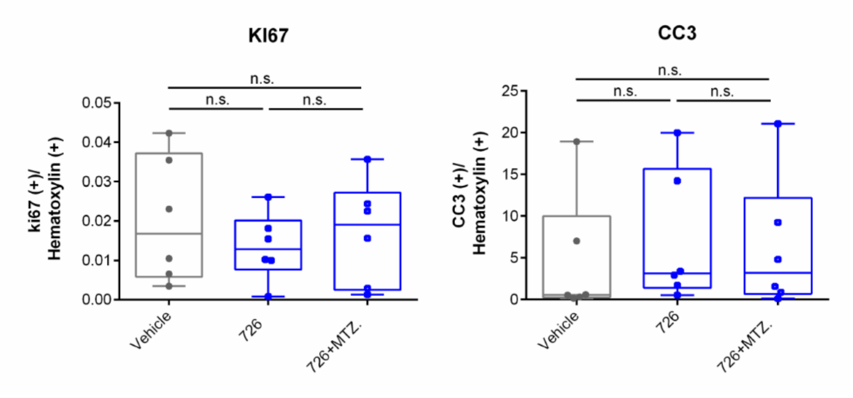

Supplementary Figure 1. Proliferation and apoptosis levels in *F. nucleatum* – infected and non-infected AT3 mouse mammary tumors. $1x10^6$ AT3 cells were injected to the mammary fat pad of 6-7 weeks old female C57BL/6 mice. When tumor volume reached 500 mm^3, mice were IV injected with PBS vehicle or with $5x10^7$ *F. nucleatum* ATCC 23726 (726). At day 8 post injection, mice were sacrificed, tumors were harvested and sectioned were stained for Ki67 or CC3 positive cells. n.s. – non-significant, one tailed, unpaired Mann Whitney test. n = 6 per group. Boxplot whiskers represent extrema, box bounds represent upper and lower quartiles, and center-line represents the median value. Source data are provided as a Source Data file.

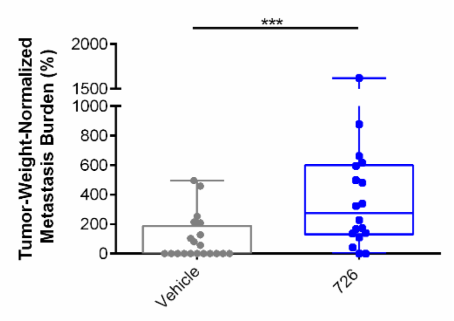

Supplementary Figure 2. Metastasis rank normalized by tumor weight. As presented in Figure 5, 1x10^6 AT3 cells were injected to the mammary fat pad of 6-7 weeks old female C57BL/6 mice. When tumor volume reached 500 mm^3, mice were IV injected with PBS vehicle or with 5x10^7 *F. nucleatum* ATCC 23726 (726). At day 8 post injection, mice were sacrificed, lungs were harvested and lung metastases were counted.

Metastasis rank was normalized by tumor weight as follows: for each mice the number of metastases per lung area ($pixel^2$) was divided by the weight (g) of the paired tumor. This was called the metastasis burden. The metastasis burden was then normalized as percent of the average metastasis burden of the sham infected (vehicle) group (set as 100% metastasis burden). Each symbol represents one mouse. Boxplot whiskers represent extrema, box bounds represent upper and lower quartiles, and center-line represents the median value of 3 combined independent experiments. Vehicle, n=20. *F. nucleatum* treated, n=18. *** p=0.001, two tailed Mann Whitney test. Source data are provided as a Source Data file.

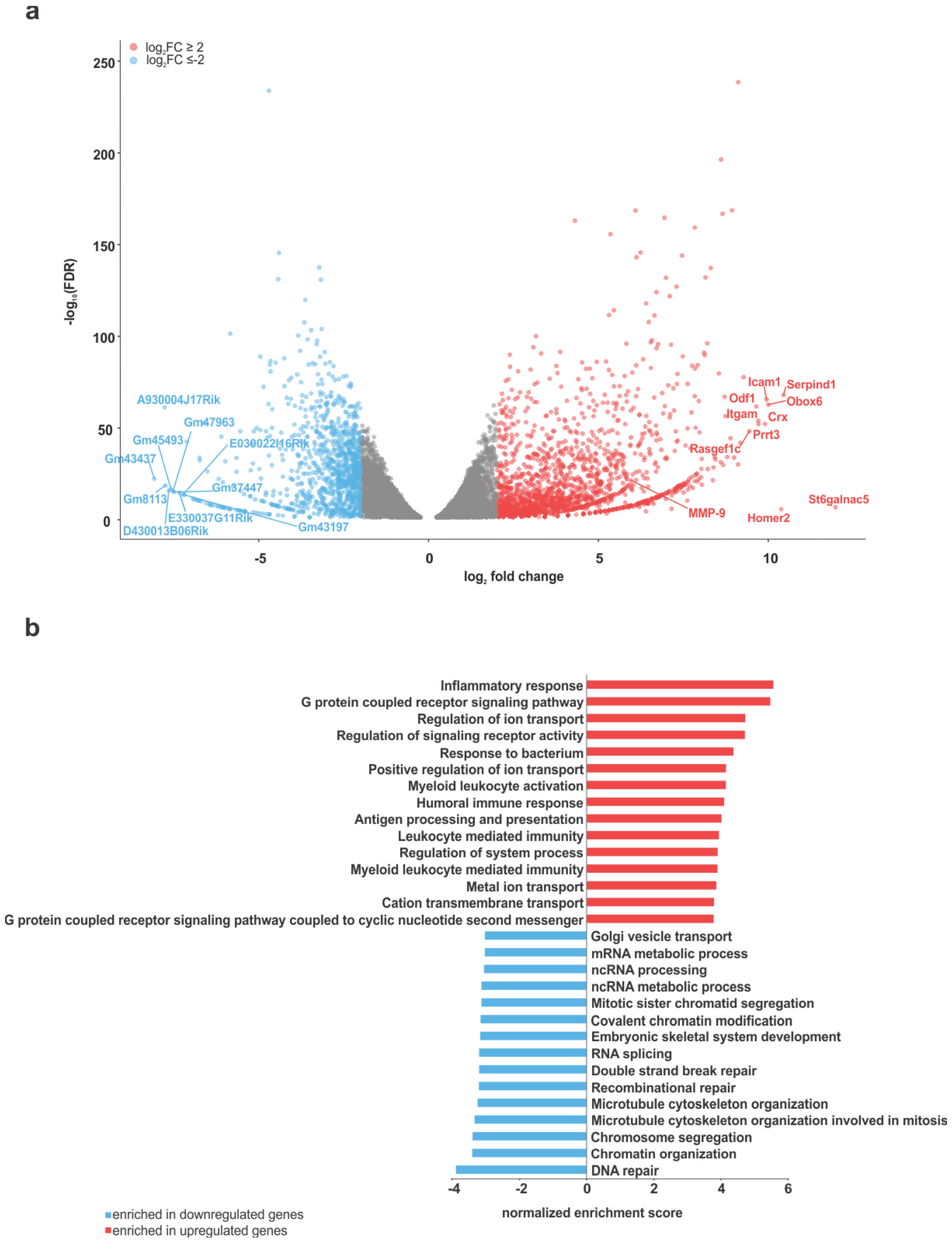

Supplementary Figure 3. Gene expression changes induced upon infection with *F nucleatum* in AT-3 cells. (**a**) Differential expressed AT-3 genes (FDR ≤ 0.05; 3 biological replicates) between cells incubated for 24h with *F. nucleatum* ATCC 23726 (MOI 1:10) and sham control. The 10 most differentially up- or down regulated genes are labeled while the complete list can be found in Table S1. Additionally, MMP-9 was indicated while MMP-2 was not detected. (**b**) Top 15 of the most enriched GO terms from the GSEA analysis upon infection with *F. nucleatum* compared to control (the complete list can be found in Table S2). Source data are provided as a Source Data file.

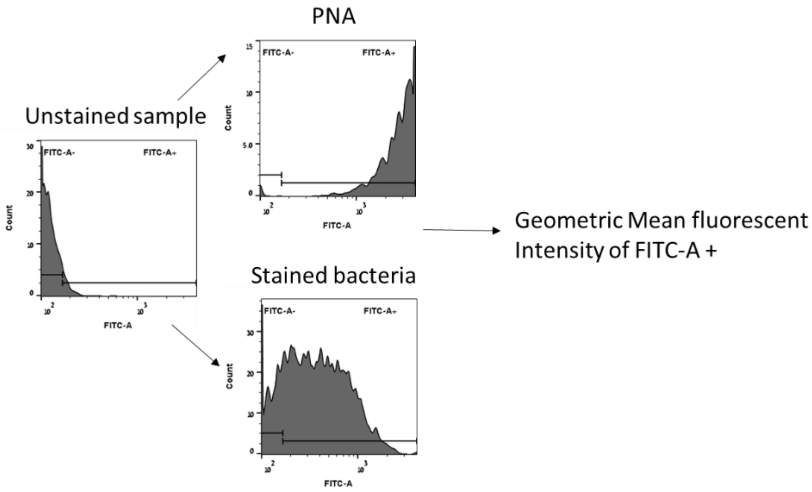

Supplementary Figure 4. Gating strategy for cell staining with PNA and FITC-labeled *F. nucleatum*. Threshold determined by unstained cells was used to analyze attachment of FITC-PNA and of FITC-labeled *F. nucleatum* to the tested cells according to FITC positive singlets. The above strategy was used in Figure 3b to quantify attachment of FITC-labelled PNA and of FITC-labeled *F. nucleatum* ATCC 23726 (and its Fap2-inactivated isogenic mutants K50 and D22) to breast cancer cell lines 4T1, AT3, MCF-7 and mouse melanoma cell line B16 used as a control, in the absence or presence of increasing amounts of soluble GalNAc.

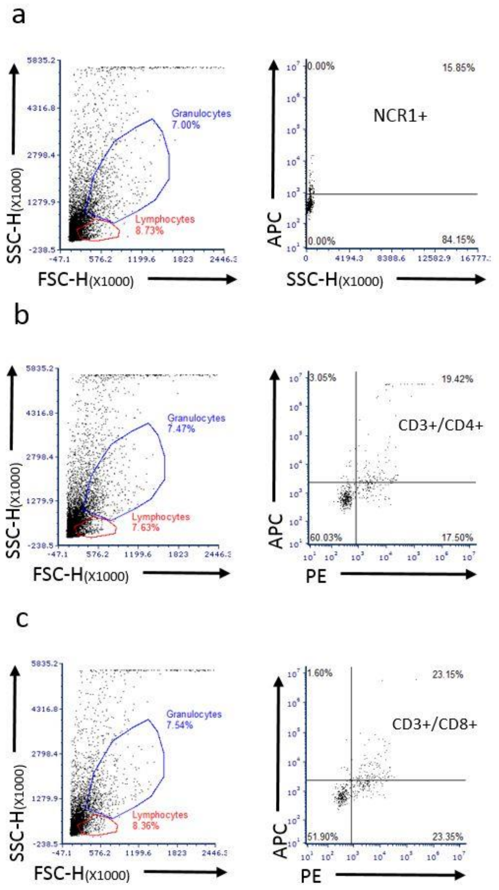

Supplementary Figure 5. Gating strategy for quantifying the abundance of NK cells, CD4+ T cells and CD8+ T cells in mouse mammary tumor. Singlets were identified and further gated as lymphocytes based on SSC-H/FSC-H distribution. Live cells were then gated as follows: NK cells were gated according to NCR1+ (a), T cells were gated according to either CD3+/CD4+ or CD3+/CD8+ (b-c respectively). The above strategy was used profile immune cells in AT3 tumors of C57BL/6 mice infected or not with *F. nucleatum* ATCC 23726. The corresponding results are presented in Figure 6a.

Supplementary Table 1
Primers used in this study

Name	Forward	Reveres	FAM Probe
F. nucleatum ATCC 237726 nusG	5'-CAACCATTACTTTAACTCTACCATGTTCA 3'	5' ATTGACTTTACTGAGGGAGATTATGTAAAAATC 3'	5'- /56-FAM/TCAGCAACT/ZEN/TGTCCTTCTTGATCTTTAAATGAACC/3IABkFQ/ -3'
F. nucleatum K50 catP	5'-GAAGGTTGACCACGGTATCAT3'	5'-CGCAACGGTATGGAAACAATC-3'	5'-/56- FAM/ATGGAAGGA/ZEN/AAGCCAAATGCTCCG/3IABkFQ/-3'
Mouse Gapdh	5'-AATGGTGAAGGTCGGTGTG-3'	5'-AATGGTGAAGGTCGGTGTG-3'	5'-/56- FAM/TGCAAATGG/ZEN/CAGCCCTGGTG/3IABkFQ/-3'
Human Gapdh	5’- TGAGTGTGGCAGGGACT-3’	5’- AGGGTGGTGGACCTCAT-3’	5’- /56FAM/CAGCAAGAG/ZEN/CACAAGAGGAAGAGAGA/3IABkFQ/-3’
P. gingivalis ATCC 33277 ISPg1	5'-ACGGACAACCTGTTTTTGATAATCCT-3'	5'-CGCAGACGACAGAGAAGACA-3'	5'-/56-FAM/TCCGCCTCG/ZEN/CTCCGAT/3IABkFQ/-3'
16S amplification and deep sequencing	F1-TGGCGAACGGGTGAGTAA	R1-AGACGTGTGCTCTTCCGATCTCCGTGTCTCAGTCCCARTG	
	F2-ACTCCTACGGGAGGCAGC	R2-AGACGTGTGCTCTTCCGATCTGTATTACCGCGGCTGCTG	
	F3-GTGTAGCGGTGRAATGCG	R3-AGACGTGTGCTCTTCCGATCTCCCGTCAATTCMTTTGAGTT	
	F4-GGAGCATGTGGWTTAATTCGA	R4-AGACGTGTGCTCTTCCGATCTCGTTGCGGGACTTAACCC	
	F5-GGAGGAAGGTGGGGATGAC	R5-AGACGTGTGCTCTTCCGATCTAAGGCCCGGGAACGTATT	
Barcodes and Illumina adaptors	FF1AATGATACGGCGACCACCGAGATCTACACTCTTTCCCTACACGACGCTCTTCCGATCTTGGCGAACGGGTGAGTAA	CAAGCAGAAGACGGCATACGAGAT-NNNNNNNN-GTGACTGGAGTTCAGACGTGTGCTCTTCCGATCT	
	FF2-AATGATACGGCGACCACCGAGATCTACACTCTTTCCCTACACGACGCTCTTCCGATCTACTCCTACGGGAGGCAGC		
	FF3-AATGATACGGCGACCACCGAGATCTACACTCTTTCCCTACACGACGCTCTTCCGATCTGTGTAGCGGTGRAATGCG		
	FF5-AATGATACGGCGACCACCGAGATCTACACTCTTTCCCTACACGACGCTCTTCCGATCTGGAGCATGTGGWTTAATTCGA		
	AATGATACGGCGACCACCGAGATCTACACTCTTTCCCTACACGACGCTCTTCCGATCTGGAGGAAGGTGGGGATGAC		
