# Supplementary material for: Breast cancer colonization by Fusobacterium nucleatum accelerates tumor growth and metastatic progression
Source: Nat Commun. 2020 Jun 26;11:3259. doi: 10.1038/s41467-020-16967-2 (PMC7320135; doi:10.1038/s41467-020-16967-2)
Supplement: Supplementary file 2 — Description of Additional Supplementary Files [file 41467_2020_16967_MOESM2_ESM.docx]

**Description of Additional Supplementary Files**

**File name:** Supplementary Data 1

**Description:** Results of the differential gene expression analysis performed using edgeR

**File name:** Supplementary Data 2

**Description:** GSEA analysis for enriched GO terms by of AT3 cells incubation with F. nucleatum
